# Anti-Mullerian Hormone as a Marker of Ovarian Reserve and Function

**DOI:** 10.7759/cureus.29214

**Published:** 2022-09-15

**Authors:** Sudwita Sinha, Amrita Sharan, Sangeeta Sinha

**Affiliations:** 1 Obstetrics and Gynaecology, All India Institute of Medical Sciences, Patna, IND; 2 Obstetrics and Gynaecology, Patna Medical College, Patna, IND

**Keywords:** ovarian volume, reproductive ageing, antral follicle count, amh, ovarian reserve tests

## Abstract

Background: Ovarian reserve tests are required to screen women with a diminished ovarian reserve so that women who are more likely to exhibit poor response to ovarian stimulation and a lower likelihood of becoming pregnant with treatment can be identified.

Aim and Objectives: This study aimed to determine whether serum anti-Mullerian hormone (AMH) level is a better predictor of ovarian reserve and function than other biochemical tests for ovarian reserve. The primary objective of this study was to find out the correlations of day 3 serum luteinizing hormone (LH), follicle-stimulating hormone (FSH), estradiol, inhibin B, AMH, ovarian volume, and antral follicle count (AFC) with advancing age; and a secondary objective was to find out the correlations between day 3 serum AMH, LH, FSH, estradiol, and inhibin B levels with AFC.

Methods: This was a prospective, single-center, observational study. A total of 100 infertile women who attended the Gynecology outpatient department over a period of two years and met the inclusion criteria were included in the study. History, clinical examination, routine investigations, hysterosalpingography for tubal patency, estimation of day 3 serum AMH, LH, FSH, estradiol and inhibin B, measurement of ovarian volume, and AFC were done. Correlations of different parameters with advancing age and with AFC were found using Spearman’s rho correlations. p-value < 0.05 was considered significant.

Results: The maximum infertile women were in the age group of 21-30 years (58 of 100). Serum AMH showed the strongest negative correlation (r=-0.931) with age, followed by AFC (r=-0.884), ovarian volume (r=-0.876), and inhibin B (r=-0.878), whereas serum LH, FSH, and estradiol showed a positive correlation (r=0.589, 0.408, and 0.638 respectively). Serum AMH also showed a strong positive correlation (r=0.972) with AFC followed by ovarian volume (r=0.919) and inhibin B (r=0.769), whereas serum LH, FSH, and estradiol showed a negative correlation (r=-0.504, -0.663, and -0.543 respectively) with AFC.

Conclusion: Among all the different tests of ovarian reserve, serum AMH was the most reliable indicator of reproductive aging and decline in the ovarian pool as well as very closely related to AFC, which is one of the best predictors of ovarian reserve.

## Introduction

Despite being gifted with a decent ovarian reserve at birth, there is an age-related decline in the ovarian reserve from mid-life onwards. Also, the risk of age-related involuntary infertility has increased in modern times. In such situations, the ability to measure reproductive aging, i.e. to tell the time on the biological clock help women decide about their plans of reproduction [1]. Ovarian reserve, the functional potential of the ovary, reflects the quantity and quality of oocytes at any given time and constitutes the resting, non-growing ovarian primordial follicle population [1]. The biochemical changes associated with age-related decline in the ovarian pool constitute a rise in follicle stimulating hormone (FSH) levels, unchanged luteinizing hormone (LH) levels, and a decrease in inhibin B and anti-Mullerian hormone (AMH) levels [1]. Ovarian reserve tests are used to screen women with diminished ovarian reserve [1]. Biochemical tests of the ovarian reserve include basal measurements, such as FSH, estradiol, inhibin B, and AMH, and provocative tests, such as the clomiphene citrate challenge test [1]. Ultrasonographic measures of antral follicle count (AFC) and ovarian volume are also tests for ovarian reserve [1]. Numerous efforts have been made to assess ovarian reserve. Previously, a composite test was used consisting of serum levels of FSH, inhibin B, and estradiol (E2) in the early follicular phase [2]. Early antral follicles produce inhibin B and E2 in response to FSH, having the classical pituitary-gonadal axis feedback loop [2]. Their serum levels are not independent of each other as they are part of a feedback system, and hence, need to be measured collectively [2]. Their levels vary widely by assay, laboratory, population, and reproductive aging, and hence separately they are poor predictors of ovarian reserve [2]. So far, the number of antral follicles (follicles of 2-10 mm size) in the ovary by ultrasonography on day 3 of the menstrual cycle, AFC best predicts ovarian reserve quantitatively [2]. A better, time-independent parameter to assess ovarian reserve is serum AMH [2]. 

Anti-Mullerian hormone or Mullerian inhibiting substance, a glycoprotein hormone of molecular weight 140 kDa, is produced by granulosa cells of preantral and small antral follicles from 36 weeks of intrauterine life until menopause [1-2]. It is a member of the transforming growth factor-β superfamily [1]. AMH derived its name from its role during male sex differentiation by inducing the regression of the Müllerian ducts [3]. It inhibits the recruitment of primordial follicles as well as growing follicles’ response to FSH [1]. Its levels progressively decline with age and are gonadotropin independent with little variation between cycles [1]. The highest production is in the preantral and small antral stages (≤ 4 mm diameter) of folliculogenesis although it is first made in the primordial follicle stage [2]. During these stages, as the follicles are microscopic, they cannot be counted by ultrasound [2]. Production of AMH gradually decreases with further growth of the follicle and finally stops once the follicle reaches 8-mm diameter [2]. The normal serum AMH level range is 2-6.8 ng/mL (14.28-48.55 pmol/L) in any phase of the cycle [2].

The number of growing follicles recruited reflects the number of primordial follicles [3]. As there is no serum marker that can directly measure the number of primordial follicles, currently the best proxy for the quantitative aspect of the ovarian reserve is a marker that reflects the growing follicles [3]. To date, AMH is best known for assessing the “functional ovarian reserve,” as it reflects the pool of growing follicles that has the potential to ovulate [3]. Based on initial studies which showed that AMH levels strongly correlated with growing follicles number, serum AMH was rapidly put forward as an indirect marker for the ovarian reserve despite limited knowledge of factors that regulate ovarian AMH expression and lack of standardized AMH assays [3]. Since serum AMH is only an indirect marker, this has led to misinterpretation of the term ovarian reserve [3]. To make a clear distinction -- the pool of resting primordial follicles is an ovarian reserve whereas the pool of growing follicles is a functional ovarian reserve (FOR) [3]. Recent studies suggest that serum AMH levels reflect FOR at all ages, and may also reflect the ovarian reserve only at older reproductive age [3].

Recent studies being conducted also suggest that AMH levels increased from birth onward to plateau at the approximate age of 25 years [3]. From age 25 years onward, it starts to decline till it reaches undetectable levels at menopause, after which, a negative correlation between AMH levels and age can be observed [3]. However, large interindividual and intraindividual variation exists for AMH levels which may be due to different ethnicity [3].

While initial studies suggested that AMH levels are independent of the menstrual cycle, recent research suggests that AMH levels show significant intracycle variation [3]. This suggests that a single AMH measurement may lead to an inaccurate assessment of the FOR leading to clinical consequences in infertility patients. Serum AMH levels may predict the risk of ovarian hyperstimulation syndrome or poor response in ovulation stimulation protocols and may predict the age of menopause [3]. Although serum AMH remains the preferred ovarian reserve marker, direct comparison between AMH values obtained from different assays is still problematic due to the lack of an international standard [3]. The development of standardized AMH cut-off values needed to enhance patient safety and prevent misinterpretation by clinicians is limited by the absence of uniformly calibrated assays [3]. Therefore, more research is required to allow proper comparison of the different assays, using larger, clearly defined age-stratified cohorts [3]. It is also yet to be determined whether the measurement of different AMH isoforms has improved clinical relevance over total AMH (current assays). Furthermore, endogenous and exogenous factors that influence serum AMH levels (hormonal contraceptive used -- type, duration; timing of measurement during the menstrual cycle, AMH assays used, BMI, leptin, vitamin D) are still not known, which limits the proper interpretation of AMH values in a clinical setting [3]. Thus, when counseling women on FOR based on AMH levels, these factors should be kept in mind. Recent studies also suggest that AMH may not follow a uniform decline trajectory with age [3]. The decline rate is found to be dependent on age, thus raising the question of at what age and how frequently AMH should be measured to predict the age of menopause accurately. Based on current studies, the use of serum AMH to predict the age of menopause still remains controversial. It is also unclear whether current results obtained in regularly cycling women can be generalized to infertile women [3]. Application of ovarian reserve test as a predictor of ongoing pregnancy seems limited as they only represent the quantitative aspect, whereas pregnancy is also dependent on the oocyte quality [3-4]. To summarize, the role of serum AMH levels as a marker for the FOR is strengthened due to the improved sensitivity and automation of AMH assays [3]. The AMH levels may be useful in predicting ovarian response to controlled ovarian hyperstimulation (COH) and age of menopause (natural or iatrogenic) [3]. However, more knowledge is still needed on endogenous and exogenous factors that regulate AMH expression for its proper interpretation [3]. Heterogeneity in AMH trajectories also limits its application in patient counseling [3]. An international standard for AMH is urgently needed to establish assay-independent cut-off values as differences exist in available assays [3].

Despite the above uncertainties regarding AMH, it is still being used as a common marker to assess ovarian reserve almost replacing the previous biochemical tests of ovarian reserve. This study aimed to determine whether serum AMH level is a better predictor of ovarian reserve and function than other biochemical tests for ovarian reserve. The primary objective of this study was to find out the correlations of day 3 serum LH, follicle stimulating hormone (FSH), estradiol, inhibin B, AMH, ovarian volume, and AFC with advancing age; and a secondary objective was to find out the correlations between day 3 serum AMH, LH, FSH, estradiol, and inhibin B levels with AFC.

## Materials and methods

Study design

This was a prospective observational study conducted over a period of two years.

Study participants

A total of 100 infertile women attending the Gynecology outpatient department over this period were asked to participate in this study after taking written informed consent.

Participants recruitment

The criteria for inclusion were reproductive age group of 18-43 years, history of infertility, having regular menstrual cycles, normal BMI, patent tubes on hysterosalpingography, adequate visualization of ovaries in transvaginal sonography, on no current hormone therapy, and no current or past diseases affecting ovaries or gonadotropin or sex steroid secretion, clearance or excretion.

Interventions

History, clinical examination, and routine investigations were done. Cases with endocrinological disorders, abnormal liver, and kidney function tests were excluded from the study. Hysterosalpingography was done for tube patency. Serum AMH, LH, FSH, estradiol, and inhibin B levels were estimated from venous samples taken on the third day of the menstrual cycle at 0900 h. Serum AMH estimation was done by sandwich enzyme immunoassay. Serum FSH, LH, and estradiol levels were measured using a solid-phase, two-site, chemiluminescent immunometric assay. Inhibin B was estimated by the enzyme-linked immunosorbent assay method. Transvaginal sonography was done on the third day of the menstrual cycle, using a 7.5 MHz transvaginal probe to measure ovarian volume and AFC. AFC was done by counting all follicles of 2-10 mm size in both ovaries and taking the sum, with normal being more than 12. The volume of the ovaries was assessed by measuring its diameter in three perpendicular directions and applying the equation of ellipsoid volume and taking the sum of both ovaries (normal: 9-11 cc).

Outcome

Correlations of all the different parameters with age and AFC were estimated. A p-value less than 0.05 was considered significant.

Statistical analysis

Sample Size Estimation

According to a study done by Dayal et al., we have taken the correlation of SAMH with different age groups and the closest means are taken to find the maximum sample size for the study, that is the age group of 21-30 years and the age group of 31-40 years. Taking 95% confidence interval and 90% power, the ratio of sample size between two groups is 1, the sample size is 100 [2].

The SPSS version 21.0 (IBM Corp., Armonk, NY) was used for the statistical analysis of data. The one-way analysis of variance (ANOVA) and Kruskal-Wallis test were used for data analysis. Post hoc comparisons were determined using the Bonferroni test, Spearman’s rho correlations, and multiple linear regression analysis. The p-value < 0.05 was considered significant, 95% confidence interval was used.

Ethical considerations

Ethical approval was obtained from Institutional Review Board, PATNA MEDICAL COLLEGE (letter no- OBG/2499, dated-02/12/2016).

## Results

A total of 100 women were included in the study. They were divided into four groups according to age, with maximum infertile women (58 of 100) in the age group of 21-30, 17 in the age group 31-40, 15 in the age group <20, and 10 in the age group >40 years.

Table 1 summarizes the correlations between the mean values of different tests for ovarian reserve and different age groups, where serum AMH is more robustly correlated with age compared to the other parameters.

**Table 1:** Correlations of different parameters with different age groups. LH, luteinizing hormone; FSH, follicle stimulating hormone; AFC, antral follicle count; AMH, anti-Mullerian hormone

Parameters	≤20 years	21-30 years	31-40 years	≥40 years	Correlation (r)
Serum LH	4.02 ± 0.31	6.37 ± 2.63	9.24 ± 9.23	28.24 ± 18.81	0.589, p < 0.05
Serum FSH	4.96 ± 0.22	6.77 ± 5.39	14.68 ± 16.11	17.31 ± 16.51	0.408, p < 0.05
Serum estradiol	19.80 ± 4.86	44.76 ± 15.73	60.76 ± 13.96	149.60 ± 93.04	0.638, p < 0.05
Serum inhibin B	88.67 ± 3.12	77.86 ± 3.73	67.29 ± 1.96	57.10 ± 6.21	-0.878, p < 0.05
AFC	16.27 ± 1.83	13.22 ± 2.29	7.88 ± 2.47	5.20 ± 1.62	-0.884, p < 0.05
Ovarian volume	9.73 ± 0.69	8.11 ± 0.45	6.91 ± 0.69	6.17 ± 0.48	-0.876, p < 0.05
Serum AMH	5.48 ± 0.29	4.01 ± 0.84	1.72 ± 0.96	0.23 ± 0.23	-0.931, p < 0.0001

A positive association (r=0.589) was found between serum LH and age suggesting that serum LH increased with increasing age. Serum FSH also showed a positive association (r=0.408) with advancing age showing that serum FSH increased with advancing age. A positive association (r=0.638) was observed between serum estradiol and age indicating that serum estradiol increases with increasing age. There was a negative association (r=-0.878) between serum inhibin B and age indicating a decrease in serum inhibin B with advancing age. A negative association (r=-0.884) was seen between AFC and age implicating the age-related decrease in the antral follicle pool. A negative association (r=-0.876) was also seen between ovarian volume and age implicating that ovarian volume reduced with advancing age. A strong negative association (r=-0.931) was seen between serum AMH and age indicating that serum AMH decreases with advancing age. This strong association between serum AMH and age signifies that AMH is a better marker of the age-related decline in the ovarian follicular pool, compared to the other parameters in this study such as serum LH, FSH, estradiol, and inhibin B.

Table 2 summarizes the correlations between the mean values of different tests for ovarian reserve and AFC. It is seen that AMH is again more strongly related to AFC.

**Table 2:** Correlation of different parameters with AFC. LH, luteinizing hormone; FSH, follicle stimulating hormone; AFC, antral follicle count; AMH, anti-Mullerian hormone

Parameters	AFC: <4	AFC: 4–7	AFC: 8–12	AFC: >12	Correlation (r)
Serum LH	19.27 ± 20.9	22.9 ± 19.1	6.66 ± 2.30	5.87 ± 2.81	−0.504, p < 0.05
Serum FSH	51.20 ± 5.39	18.57 ± 14.72	6.69 ± 0.28	5.54 ± 0.49	−0.663, p < 0.05
Serum estradiol	133.33 ± 130.48	108.77 ± 80.42	53.40 ± 14.79	38.52 ± 19.11	−0.543, p < 0.05
Serum inhibin B	67.00 ± 2.64	61.08 ± 9.39	72.48 ± 6.32	80.58 ± 6.01	0.769, p < 0.05
Ovarian volume	5.33 ± 0.25	6.34 ± 0.37	7.49 ± 0.38	8.65 ± 0.76	0.919, p < 0.05
Serum AMH	0.03 ± 0.02	0.31 ± 0.24	2.77 ± 0.67	4.63 ± 0.62	0.972, p < 0.0001

It was observed that serum LH decreased with increasing AFC as evident by the negative association (r=-0.504). Serum FSH was also seen to decrease with increasing AFC with a negative association (r=-0.663). Similar to LH and FSH, serum estradiol also showed a negative association with AFC (r=-0.543) indicating that serum estradiol decreased with increasing antral follicle pool. However, serum inhibin showed a positive correlation with antral follicle pool (r=0.769) meaning it increased with increasing AFC. Similarly, the ovarian volume also increased with increasing AFC as evident by the positive association (0.919). Serum AMH, like ovarian volume, showed a strong positive correlation (r=0.972) with AFC suggesting that serum AMH is closely associated to AFC and ovarian reserve.

## Discussion

This study was done on 100 infertile women to compare the association of AMH and other biochemical parameters with age and AFC. The upper limit of age was taken as 43 years since this study was conducted in the state of Bihar, India where early marriage and childbearing is still common and women seeking workup of infertility above the age of 40 are hardly encountered in clinical practice.

The study was conducted to determine whether serum AMH levels can be used to predict ovarian reserve and function by evaluating the direct relationship between peripheral AMH levels and the ovarian follicular status on the third day of menstruation and comparing the strength of correlations between the number of AFC and hormonal parameters implicated directly or indirectly in the eventual stages of follicle genesis. Age-dependent loss of fertility due to decreasing follicle pool has been described. However, this fact may vary individually. Serum FSH level on day 3 increased with the advancing age. Serum estradiol level was also found to increase with increasing age. Though these results may look to be contradicting the fact of the negative feedback loop, it can be explained by the fact that serum estradiol level basically depicts follicular growth rather than the number of antral follicles [2]. Day 3 serum estradiol levels are typically higher in older women with advanced reproductive aging in response to an elevation in FSH and a decrease in inhibin B resulting in advanced follicular growth at the end of the preceding luteal phase [2]. Serum AMH levels had a highly significant reduction with the increasing age (p < 0.0001). A noticeable reduction in the number of early antral follicles in women with low AMH characterizes the decline of ovarian function that results from relative follicular attrition [2]. Dayal et al. found that with advancing age -- serum AMH level, AFC, ovarian volume, and inhibin B decreased; and serum FSH, LH, and estradiol increased which were similar to the findings in our study [2]. The present study was designed to evaluate the direct relationship between the ovarian follicular status and peripheral AMH levels on day 3 of menstruation and to compare the strength of correlations between the hormonal parameters and the number of AFC. It was observed that serum AMH levels were closely related to early AFC, with a remarkably more intense relationship than those obtained with serum levels of inhibin B, estradiol, FSH, and LH. These results corroborate to the findings reported previously by other investigators. Patients who are destined to fail can be identified by tests of ovarian reserve. In the general infertile population, age as a marker of ovarian function was found to have a prognostic value because age showed a strong correlation with all the markers of ovarian function like AMH, AFC, estradiol, and FSH. AMH can screen ovarian function in the general sub-fertile population as it has a role in both the processes of initial and cyclical recruitments [2]. It reflected the continuous decline of the follicle pool with age better than the other markers, hence, it appears to be the best marker of gradual dwindling of follicle numbers and ovarian volume and the most reliable reflection of reproductive aging. van Rooij et al. also found that serum AMH levels were highly correlated with the number of antral follicles (r=0.77; p < 0.01) and the number of oocytes retrieved (r=0.57, p < 0.01) [4].

The advantage of the use of AMH over AFC is that ultrasound is not needed. Furthermore, AMH levels do not change in response to gonadotrophins and thus can be measured throughout the cycle in contrast to the other parameters giving an advantage for both patients and clinicians [4]. Fluctuation in serum AMH levels during the menstrual cycle is also very small (Cook et al., 2000), supporting the feasibility of its assessment throughout the cycle [4-5]. Tremellen et al. concluded that when estimating ovarian reserve, AMH should be used as an adjunct to FSH or estradiol or AFC [6]. de Vet et al. also found that AMH concentrations correlated with age, the number of antral follicles and with FSH to a lesser extent but not with inhibin B levels [7]. Elgindy also opined that mid-luteal and early follicular AMH may offer good prognostic value for clinical pregnancy [8]. Panchal and Nagori suggested that AFC alone is sufficient for estimating ovarian reserve [9]. Jain et al. found a significant correlation between AMH and AFC. AMH increased with age till the third decade of life and showed a negative correlation with AFC after which, AMH started decreasing with age and showed a positive correlation with AFC [10]. Suardi et al. found a negative correlation between serum AMH and an ovarian volume containing endometrioma although it was not statistically significant [11]. Anuradha et al. concluded that AMH is considered the most reliable investigation for ovarian reserve but is costlier than comparatively low-cost AFC and hence, AFC can be done in poor patients for ovarian reserve test [12]. This will be applicable particularly in settings with low socioeconomic status with low family income. Fanchin et al. showed that AMH levels gradually decline during multiple follicle maturation in COH probably reflecting the drastic reduction in small antral follicles due to COH, confirming the scarce AMH expression by larger follicles [13]. Anderson et al. found that AMH correlated with patient age and treatment gonadotoxicity and declined at a similar rate compared to healthy individuals following any post-treatment recovery [14]. Göksedef et al. concluded that serum AMH levels relate strongly with ovarian follicular status which is more significant than other ovarian reserve parameters [15]. Muttukrishna et al. showed that AMH is the best single marker of ovarian reserve and the combination of AMH with FSH and inhibin B is modestly better than the single marker [16]. Permadi et al. found a significant positive correlation between AMH (p ≤ 0.001, r=0.530), AFC (p ≤ 0.001, r=0.687), and AMH-AFC combination (p ≤ 0.001, r=0.652) [17]. Tran et al. stated that AMH has limitations because it only reflects the growing follicular pool that is responsive to gonadotropins and, therefore, may not be solely reflective of the underlying primordial pool [18]. Arvis et al. showed that the distribution of AMH and AFC was characterized by a wide dispersion of values, twice more important for AFC, and a logarithmic distribution [19]. The faster decline in AMH with age compared to AFC suggested that their correlation changes with age [20]. Reproducibility for AMH seemed much better than for AFC [19]. Comparing AMH and AFC for the prediction of ovarian response depending on the local conditions for measuring these indicators [19]. Iwase et al. found that AMH level monitoring is useful in infertility treatments, patients undergoing assisted reproductive technology, diagnosing ovarian failure, polycystic ovarian syndrome, granulosa cell tumor; evaluating iatrogenic ovarian damage, planning reproductive health management, improving prediction of pregnancy and live birth, etc. [20]. Li concluded that AFC and AMH had only modest predictive performance on the occurrence of cumulative live birth without any additional value on top of the women's age [21].

The above study showed that AFC and AMH correlated with age-related decline in ovarian reserve which was consistent with the findings of most other studies. AMH also correlated strongly with AFC as already proven by previous studies. However, the limitations of AMH should be considered before advising AMH as a single ovarian marker despite its advantages mentioned by previous studies as a lot many contradicting results have emerged in recent years. Hence, it is not worthwhile to use any of the ovarian reserve markers including serum AMH or AFC as the basis to exclude subjects from attempting assisted reproduction treatments, and when used for prognostic counseling the women should understand the limitations and imprecision of these indices in anticipating the treatment outcome. Also, AMH represents the growing follicular pool and not the resting primordial pool. Thus it represents only the quantitative aspect and not the qualitative aspect. Thus, to conclude it would not be wise to make clinical decisions based on AMH levels alone. Whenever in doubt, it should always be confirmed with AFC.

Limitations

The main limitation of this study is its small sample size. This study was conducted in a small region with patients of similar ethnicity. Thus, the findings of this study cannot be generalized to other ethnic groups or women residing in different regions. The participants in this study did not include women of the older age group in whom the age-related decline is more marked than in the younger population. Several factors influencing AMH were not taken into account which could have affected the results of the study. Study participants were all with regular menstrual cycles. As such, the generalization of the results to patients with irregular menstrual cycles seems questionable.

## Conclusions

The study revealed that with the assessment of ovarian reserve and function, we can identify those women who are destined to fail early and help them make plans about conception. Age, as an ovarian function marker, showed significant correlations with all markers of ovarian function, thus showing a prognostic value for ovarian function. AMH better reflected the age-related decrease in the follicular pool than other markers, giving the most reliable reflection of reproductive aging for any particular woman.
